# Remote Patient Monitoring and the Need for a New Care Model: A Narrative Review of Implementation Challenges

**DOI:** 10.7759/cureus.102803

**Published:** 2026-02-01

**Authors:** Goutham V Meda, Ailie H Brennan-Davies

**Affiliations:** 1 Internal Medicine, Western Health, Melbourne, AUS; 2 Internal Medicine, St Vincent's Hospital, Melbourne, AUS

**Keywords:** artificial intelligence, digital health, healthcare technology, preventive, remote monitoring, remote patient monitoring systems, telehealth

## Abstract

Remote patient monitoring (RPM) has the potential to replace reactive, clinic-based encounters with preventive, continuous care delivered in patients’ homes. However, the adoption of RPM has not kept pace with the global ascendance of telehealth in recent years. This narrative review draws on published literature on RPM since 2010, with an emphasis on barriers to RPM program adoption and implementation in clinical settings. Identified challenges to widespread adoption include data overload, ambiguous clinical responsibility, poor integration into existing workflows, and patient and device usability issues. Though the technology itself is reliable, a clearly defined clinical care model is required to maximise the potential of RPM and prevent overburdening healthcare systems. This narrative review suggests that a protocol-driven model with a tiered escalation plan may be beneficial. Future prospective implementation research is required to evaluate the use of alternative, restructured care models for delivering RPM programs.

## Introduction and background

Remote patient monitoring (RPM) is the use of digital devices and patient-reported data to collect health information in patients’ home environments. The information is used to monitor acute and chronic diseases such as heart failure [1], hypertension [2], chronic obstructive pulmonary disease (COPD), and post-discharge care. The data are sent to clinical care teams, who can act upon the information by providing advice over the phone, changing medication remotely, or requesting a clinical review. Studies have shown that RPM is beneficial for both patients and clinicians [3,4]. RPM can provide a safety net through timely detection of health issues, allowing earlier treatment to be delivered before health deterioration occurs, thereby reducing the risk of hospital readmission as well as lowering non-hospitalisation costs [3].

The use of RPM first expanded rapidly during the coronavirus disease 2019 (COVID-19) pandemic, when prompt identification of deteriorating health became critical. People with COVID-19 were at high risk of dying from unrecognised hypoxaemia detected by pulse oximetry. Due to the need to self-isolate during the pandemic and limited hospital space and staffing, RPM was used to monitor patients at home for clinical deterioration using continuous pulse oximetry measurements. A large retrospective analysis of high-risk COVID-19 patients conducted in the USA found that patients who engaged in RPM monitoring experienced significantly lower rates of mortality (0.5% vs 1.7%), lower rates of hospitalisation (13.7% vs 18%) and ICU admission, as well as reduced costs of care ($2306.33 USD vs $3565.97 USD), than patients who did not engage with RPM [5]. The benefit of RPM became clear and widely adopted during the pandemic and was utilised across the world. The importance of resource allocation when implementing RPM programs has also been highlighted. Targeting people at the greatest risk of clinical deterioration and hospitalisation has been shown to yield the greatest benefit [6].

Current applications of RPM

Since the pandemic, RPM has been widely studied in chronic disease populations. A study involving patients with heart failure found that RPM programs are linked with reductions in hospitalisations and mortality compared with usual care [1]. In hypertension, RPM has been associated with significant improvements in blood pressure control and reductions in uncontrolled hypertension [2]. Broad systematic reviews also suggest that RPM programs for cardiovascular disease and COPD may reduce hospital admissions, length of stay, and emergency department use [7]. The data collected from RPM allows clinicians to have more information when reviewing a patient, thereby enhancing clinical decision-making. Patient engagement and satisfaction can also improve with better visibility into their health conditions. RPM programs can be used for an array of clinical conditions, and the benefits to both patients and care providers are diverse when integrated into a well-structured care model (Table 1).

**Table 1:** Practical clinical examples showing how RPM data are used to detect early change and guide timely intervention across common chronic and post-acute conditions in everyday practice RPM: remote patient monitoring; BP: blood pressure; HR: heart rate; COPD: chronic obstructive pulmonary disease

Clinical condition	Common RPM parameters	Early trends detected	Example of RPM-enabled intervention	Potential clinical impact
Heart failure	Daily weight, BP, HR, symptom scores, activity level	Gradual weight gain, rising resting HR, increasing dyspnoea	Nurse contacts patient; diuretic dose adjusted; dietary counselling reinforced	Prevents progression to overt volume overload, reducing ED visits and re-hospitalisation
Diabetes mellitus	Continuous glucose monitoring, fingerstick glucose, diet/activity logs	Rising fasting glucose, increased glycaemic variability	Medication or insulin adjusted; targeted dietary counselling	Prevents prolonged hyperglycaemia and reduces the risk of acute and chronic complications
COPD	Oxygen saturation, respiratory rate, symptom surveys, and activity level	Declining oxygen saturation, increasing dyspnoea, reduced activity	Early steroids, antibiotics, or inhaler escalation	Prevents full exacerbation and hospitalisation
Post-surgical/post-discharge Patients	HR, temperature, activity level, symptom reporting	Rising HR or temperature, declining mobility	Early clinic visit or treatment for infection or complications	Reduces readmissions, sepsis, and delayed complication detection
Mental health (depression, bipolar disorder)	Sleep patterns, activity level, self-reported mood, app engagement	Reduced sleep, social withdrawal, increased activity (mania)	Early psychiatric outreach or medication adjustment	Prevents relapse, hospitalisation, or crisis events

## Review

Methodology

This narrative review was developed through a targeted, non-systematic review of the published literature, focusing on the challenges associated with adopting RPM programs. Relevant articles were identified using PubMed and Google Scholar, with the inclusion criteria consisting of articles written in English, available in full text, and including peer-reviewed trials, reviews, and reports that examined implementation challenges of RPM rather than device performance itself. Research articles published between 2010 and 2025 were included to ensure the currency of the research findings. As a narrative review, this approach may not capture all available studies and does not formally assess study quality or the risk of bias.

Challenges of implementing RPM

Despite its considerable promise, RPM has produced inconsistent clinical and economic outcomes when implemented in real clinical practice. Generally, there has been little concern about the quality of the data produced in RPM protocols. Modern RPM platforms tend to provide consistent physiologic measurements with reliable online transfer of data [8,9]. Instead, the bottleneck lies downstream - at the level of clinical interpretation, workflow integration, and actionable response. The following themes focus on the main, recurring issues identified during the literature search for implementation challenges to remote patient monitoring adoption.

Data Overload and Alert Burden

In many current implementations, RPM generates large volumes of continuous data and alerts that must be reviewed by clinicians who are already operating at or beyond capacity. A study by Hailu et al., collecting qualitative data from physicians, found that many participants frequently had to work additional hours to review data. Participants also specifically reported difficulty reviewing the data during their working hours due to losing focus on the patient being seen concurrently, highlighting a potential patient safety risk [10].

A retrospective cohort study researched the alert burden in a multi-centre cohort of more than 26,000 patients with cardiac implantable electronic devices (CIEDs) programmed with remote monitoring capabilities. The study found that over 12 months, the devices sent 205,804 remote monitoring transmissions, of which 82,797 were alerts. Although this study provides data only on a very specific device type (CIEDs), the researchers conclude that current teams are being overwhelmed by the vast volume of data transmissions [11]. Another study of the efficacy and safety of RPM for Implantable Cardioverter-Defibrillator found that only 6.6% of data received required clinical action such as reprogramming, medication changes, and lead/system revision [12].

Abdolkhani et al. performed in-depth interviews with healthcare providers, health information professionals, and commercial providers of RPM solutions to ascertain the major challenges in receiving patient-generated health data. Healthcare professionals stated that challenges with data overload were making it difficult to prioritise information. Commercial service providers mentioned that the lack of contextualisation of data reduced the meaningfulness of the report information. Furthermore, multiple candidates interviewed raised the issue that guidelines were needed to focus on the most relevant and essential data for patient care to deal with the large influx of patient-generated data being received [13].

Unclear Clinical Responsibility Over Incoming RPM Data

The large volume of incoming RPM data needs to be received, organised, prioritised, and acted upon by a member of the healthcare team. However, studies have identified that it is often unclear which member of the team is responsible for each action. A qualitative analysis was performed in 2022 to identify the effects of RPM on clinical staff workflows. The scoping review found that when introduced to a practice, RPM projects seem to underestimate the complexity of healthcare systems due to the diverse roles of the healthcare professionals involved and their needs. León et al. report that staff were frequently found uncertain of who was responsible for data review, alert response, and follow-up actions. As a result, this often leads to ambiguity in tasks and roles, producing a lack of structure in the workflow of the clinical staff [14].

In the study by Hailu et al., primary care physicians reported that RPM created a variation in who reviewed and acted on data. The solution implemented relied on clinic staff (medical assistants and nurses) to initially review the data, and then only urgent cases were flagged for review by a physician. Where staff availability allowed this, this system of escalation was beneficial and proved an effective method to deal with the high volume of data [10].

O’Shea et al. conclude in their study of RPM alerts that the large number of transmissions from the CIED devices highlights the need for new management pathways. The researchers propose that summarisation of alerts by qualified cardiac technicians combined with automation and artificial intelligence may help clinicians to focus on true positive alerts and streamline workflow [11].

The legal standpoint of clinical responsibility also becomes unclear, as explained by Van Grootven et al. In their study, teams generally indicated that patients maintained some responsibility for seeking help when RPM indicated. However, overall responsibility was often attributed to the supervising medical doctors in the hospital. The lack of clarity led to doctors being concerned about legal cover, and some doctors added liability clauses onto their insurance specifically covering RPM in teleconsultations [15].

Lack of Integration and Interoperability

Tagne et al. researched the challenges for RPM programs in rural and regional areas of Australia. They found that participants repeatedly emphasised the need to integrate RPM technologies into existing healthcare systems and clinical records. As rural Australia is so vast, patients have to travel long distances to their local hospital. Therefore, challenges in sharing data between regional healthcare settings were cited as a more pertinent issue. Furthermore, poor interoperability and system integration were directly quoted as the reason for health services to reject RPM technologies entirely [16].

A review in 2024 found that RPM has not succeeded in optimising care for chronic conditions due to a care model focused on passive monitoring of medical conditions. Fudim et al. hypothesised that a shift of focus is needed where dedicated external health care teams provide holistic management on a longitudinal basis [17]. This study suggests an issue with the current care model as a whole, whereas others state issues with the integration of RPM data into electronic medical record systems.

A study looking at chronic disease management in the USA identified that RPM interoperability challenges are a core technical barrier to implementation. The authors recommend that RPM requires new approaches to clinician workflow and care delivery models to fully realise its value [18]. Other papers suggest that further research is needed to enhance data security and prevent breaches of data before integration can be achieved [19].

Patient and Device Usability Issues

Research by Van Grootven et al. investigated how patients with COVID-19 experienced RPM programs in 12 selected healthcare organisations in Belgium. Patients who did not have the necessary language or digital skills reportedly struggled with managing the RPM devices. This led to concerns about patient eligibility to use the technology as well as health equality concerns. Furthermore, patients sometimes lost trust in data because they perceived the data to be inaccurate and were less likely to take appropriate action as a result [15].

Another study identifies that there are vast differences in the usability scores of different home health care devices. Usability scores ranged from 98 (oxygen masks) to 59 (home hormone test kits). Statistically significant differences were found in the subjective usability ratings given to different devices [20].

Baumann et al. performed a literature review that illustrated significant variability in data based on the patient’s ability to use the devices. Device usability was identified as one of the main barriers to RPM applications. In response to these findings, the researchers suggest the ‘RPM usability impact model’ to guide implementation activities among patients as well as caregivers. The model suggests that patients’ ability to use RPM devices should be assessed by looking at: patients’ characteristics, patient compliance with RPM, device placement on the body, and technical device limitations [21].

Equity concerns further complicate adoption [22]. Digital literacy, language barriers, access to reliable internet, and comfort with technology vary widely across patient populations. Without tailored support, RPM programs risk disproportionately benefitting more technologically savvy patients while possibly failing to engage those at the highest clinical risk [22].

Discussion

The findings from the review suggest that current RPM programs can be limited by organisational deficiencies in managing: data overload, clinical responsibility, integration into current systems, and usability problems. Wider issues are also mentioned when RPM is introduced into systems, such as patient safety, legal issues for clinicians, and data security [10,15,19]. The studies by O'Shea et al. and Abdolkhani et al. highlight the need for clear guidelines to deal with the volume of data and unclear clinical roles [11,13]. Whereas Baumann et al. recommend a specific model to determine patients' ability to use the devices [21]. Due to the relatively new field of RPM, our findings are mostly derived from observational and qualitative studies included in this review. Paul et al. suggest that more structured research with new care delivery models is required to realise the full potential of RPM [18].

Without a care delivery framework designed to convert continuous data into structured, timely, and appropriately triaged clinical action, RPM risks becoming a high-volume data stream with limited real-world impact. This gap highlights the need for a new care model that is built with clear, protocol-driven, and team-based responsibility rather than attempting to add large volumes of RPM data to current practices of episodic, clinician-based reviews.

An Alternative Care Model

A shift towards team-based, protocol-driven, and asynchronous management is needed to progress RPM from merely acting as a technological adjunct to becoming a true driver in the transformation of care delivery. Responsibility for interpretation and response would be deliberately tiered, aligning the level of human involvement with the level of clinical risk.

**Figure 1:**
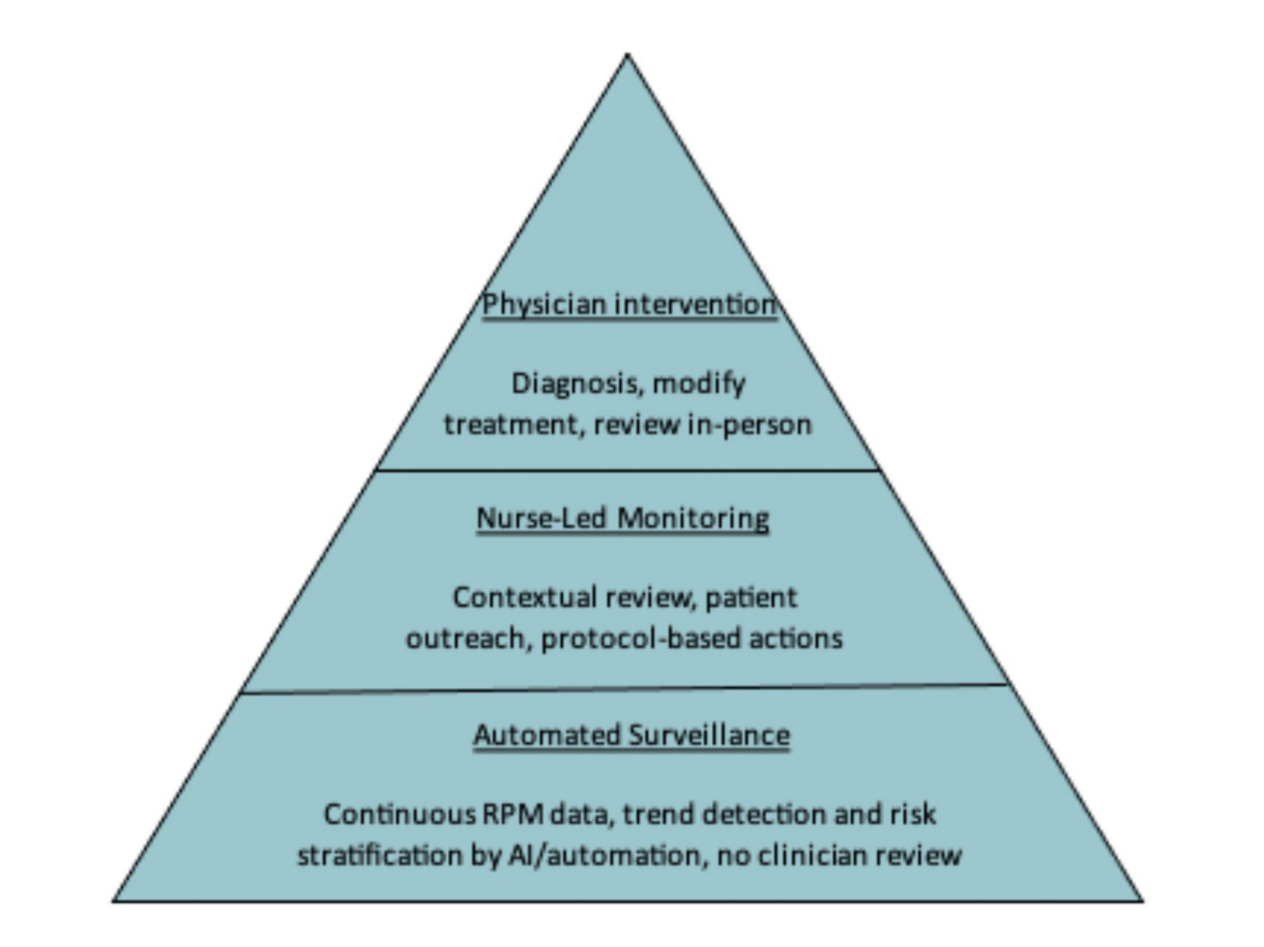
Example of hierarchical escalation structure for RPM data review RPM: remote patient monitoring

We propose an alternative care model with this framework in mind. The model can be separated into three main tiers (Figure 1). At the foundation of this model, automated analytics and AI-driven triage continuously process high-volume RPM data to filter noise, identify trends, and stratify risk. The majority of data variation is managed at this level without generating clinician alerts.

**Figure 2:**
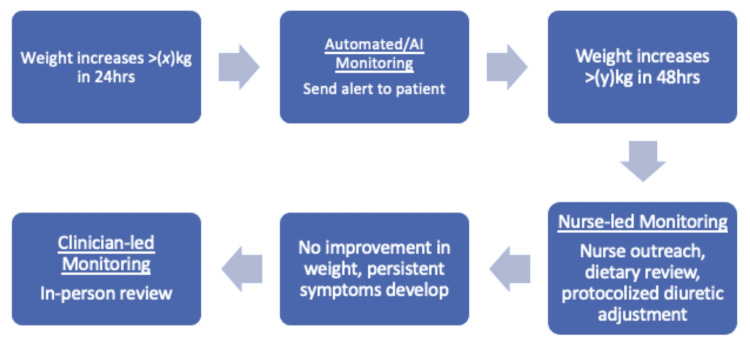
Example of each step of escalation guided by pre-set protocols in RPM of a heart failure patient RPM: remote patient monitoring

Protocols should be in place to determine action steps at each tier of the model and should indicate when to escalate to the next tier of escalation (Figure 2). Only signals that meet predefined clinical thresholds or demonstrate concerning trajectories are escalated to a nurse-led monitoring layer, where contextual assessment, patient outreach, and protocol-based interventions occur. Escalation to physicians is reserved for cases requiring diagnostic uncertainty, treatment modification, or complex clinical judgement.

Encounters remain essential, but they occur as deliberate escalation points rather than default mechanisms of care delivery. By reducing data volume at the point of capture and concentrating physician attention on high-risk exceptions, this model directly addresses alert fatigue, workforce limitations, and scalability-key barriers that have constrained the real-world impact of traditional RPM implementations.

A real-world example of this approach can be seen in the Veterans Health Administration (VHA) Home Telehealth program [23] in the USA. The program combined home monitoring with centralised nurse-led care coordination, protocol-driven escalation, and physician oversight only when thresholds were crossed. Evaluations demonstrated approximately 41% fewer hospital admissions and a 70% reduction in inpatient days of care [23]. Although their unique healthcare setup for veterans can be difficult to generalise for broad healthcare systems, the program is one of the largest and longest-running RPM deployments. Importantly, success was attributed not to the devices themselves, but to a care model explicitly designed to convert continuous data into actionable, tiered clinical responses.

Similar principles underpinned the previously mentioned successful COVID-19 RPM programs [3], where centralised monitoring teams, standardised escalation pathways, and clear ownership of responses enabled early intervention, reduced hospital utilisation, and lower mortality. These examples reinforce that RPM delivers value not when layered onto existing workflows, but when paired with a care model built for continuous data, early action following protocols, and team-based delivery.

Future directions

From Physiological to Biochemical Monitoring

To date, RPM has mainly focused on monitoring patients’ physiological changes through vital signs and reported activity or symptoms. The future lies a step beyond this with monitoring on a biochemical level. New technology is being developed that is able to continuously monitor the patient’s biochemical markers through lab-on-a-patch testing [24]. This data can then be remotely transmitted to a clinical care team for review and action.

Although these technologies are still in relatively early clinical trial phases, such devices have the potential to accelerate the scope of RPM. Biochemical markers often derange even before vital signs change or symptoms develop. Thus, allowing even earlier detection of disease progression and earlier intervention.

Future Healthcare Paradigm Shift

RPM allows care teams to oversee patients spread across a wide geographical area in a similar way to supervision in inpatient wards and nursing homes. This supervision occurs while patients remain in their own homes, preserving independence while enabling a level of clinical oversight previously limited to institutional settings. Such monitoring is especially valuable for elderly and chronic disease populations.

Increasingly, hospital-in-the-home is becoming a regular, recognised team in many hospitals. The idea of virtual wards and tele-ICU models is also being trialled and investigated. For all of these concepts to work well in practice, RPM will need to perform centre stage to deliver data remotely.

## Conclusions

RPM has demonstrated significant potential to prevent patient deterioration through preventive measures. However, adoption of RPM programs has faced challenges, including data overload, unclear clinical responsibility, poor integration into existing care pathways, and patient and device usability issues. The benefits of RPM may also vary depending on the condition, the clinical risk of the population, and the implementation model used. To realise its full value, RPM may benefit from a clearly defined clinical care model, rather than functioning as a passive data collection tool. Such a model should focus on clear, protocol-driven monitoring with tiered escalation, in which automated systems manage routine variation, and clinicians intervene only when predefined thresholds are breached. Looking ahead, RPM could evolve to incorporate continuous biochemical monitoring within care models that enable even earlier intervention. A future area of research could examine the safety and efficacy of implementing an alternative care model, such as the one described above, within an RPM program.
